# The Influence of Bullying and Cyberbullying in the Psychological Adjustment of Victims and Aggressors in Adolescence

**DOI:** 10.3390/ijerph16122080

**Published:** 2019-06-12

**Authors:** Estefanía Estévez, Jesús F. Estévez, Lucía Segura, Cristian Suárez

**Affiliations:** 1 Department of Health Psychology, Universidad Miguel Hernández de Elche, Elche, 03202 Alicante, Spain; eestevez@umh.es (E.E.); lucia.segura@goumh.umh.es (L.S.); 2 Department of Education and Social Psychology, Universidad Pablo de Olavide, 41013 Sevilla, Spain; csuarel@upo.es

**Keywords:** adolescence, bullying, cyberbullying, victim, aggressor, psychological adjustment

## Abstract

The objective of the present study was to analyze the extent to which violent peer behavior and victimization, both traditional and cybernetic, and predict certain indicators of psychological maladjustment in adolescents, such as self-concept, satisfaction with life, feeling of loneliness, depressive symptomatology, perceived stress, social anxiety, empathy, and emotional intelligence. Participants in the study were 1318 adolescents of both sexes, aged between 11 and 18 years and enrolled in Compulsory Secondary Education schools. The design of the study was cross-sectional. The results indicated that the victims generally present greater maladjustment than the aggressors. Both victims and cybervictims showed a greater decrease in all the dimensions of self-concept, compared with aggressors and cyberaggressors. However, the two types of aggressors showed a higher likelihood of presenting low levels of empathy. Feeling of loneliness, depressive symptomatology, perceived stress, and degree of life satisfaction was more probable to be present in all groups of aggressors and victims. Finally, with regard to emotional intelligence, victims had a higher probability of obtaining low scores in all the dimensions of this construct; this was the case for traditional aggressors only in the dimension of emotion regulation. These results contribute to our understanding of the consequences of harassment in the adaptation of the students involved, with relevant practical implications.

## 1. Introduction

School violence is a social problem that affects a growing number of children and adolescents at the international level [1,2,3], with significantly negative consequences for the physical and psychological health of the individuals involved [4,5]. Bullying in the educational context implies unjustified, repeated aggressive behaviors of one or several students against another classmate with the intention of harming him or her; the types of aggressive behavior can be of a verbal, physical, psychological, or relational nature [6].

Over the last few years, a new form of peer violence has emerged—cyberbullying—based on the use of information and communication technologies as the scenario of the aggressive behaviors [7]. These cyberattacks cause greater insecurity in the victim, as there are no places or moments foreign to the harassment [8]. In addition, due to the means by which the violence is carried out, it can be observed by a large number of bystanders for an indefinite number of times, which makes the potential damage even greater than that of traditional harassment [9].

### 1.1. Aggressive Behavior towards Peers and Psychological Maladjustment

The scientific literature on the relationship between aggressive behavior towards peers at school and variables of psychological adjustment has focused mainly on identifying risk factors that predispose or precipitate an intimidating and humiliating behavior toward other classmates [10]. In particular, among the risk factors identified in the adolescent stage for the development of aggressive behavior towards peers are, among others: low life satisfaction [11,12], high psychological distress [13], deficits in empathic ability [14,15], tendency to depressive symptomatology [16], and low self-esteem [17]. Taylor et al. [18] observed that aggressors have a positive self-concept in the physical (physical attractiveness) and social dimensions (popularity), but they report more negative self-perceptions in reference to the family and academic domains of self-concept.

On another hand, Liau et al. [19] found that high school students with lower emotional intelligence showed higher levels of aggressive and criminal behavior. In the same direction, other authors such as Zimmerman [20] and, more recently, [21] identified some deficits in certain aspects of emotional intelligence in adolescent aggressors, specifically in aspects of the perspective-taking of others, self-control, and social skills in general. Additionally, Bohnert et al. [22] noted that children who presented more violent behavior tended to have more difficulty controlling their anger and understanding their own emotions and their origin.

Parallel to this, with regard to cyberbullying, recent works indicate that cyberbullies show lack of empathy with their victims and a high probability of presenting addiction to new technologies [23], as well as more school absenteeism and depressive symptoms [24,25], a low degree of self-control, and high levels of feelings of frustration [26]. Another important aspect is that traditional bullying perpetration has been identified as one of the main predictors of cyberbullying [27,28], with a great overlap between the two types of peer harassment [29,30].

### 1.2. School Victimization and Psychological Maladjustment

Research on school victimization has focused primarily on identifying the consequences of being subjected to aggressive behavior and humiliations committed by peers [31]. Any type of aggression seems to negatively influence the victim’s life, which is shown in behaviors like school or institute refusal, high levels of social anxiety, depressive symptoms [32], and feelings of loneliness and stress, as well as low self-esteem and low life satisfaction in general [10,33].

Other authors like Erath et al. [34] have noted that victims of school violence often show greater difficulty in making emotional adjustments in their daily life experiences, which may eventually trigger a greater inability to take on the perspective of others, thus sometimes negatively affecting their empathic capacity [35]. In fact, in some studies have concluded that certain deficits in the capacity to express and regulate emotions may be predictors of victimization and cybervictimization [36,37]. In this sense, it seems that victims mainly use emotion-focused coping styles, which implies more attention to their own emotions and, given the complexity of their personal situation, also more difficulties to manage their emotions appropriately [36].

With regard to the victims of cyberbullying, it has been observed that their suffering and perception of insecurity can be aggravated by the lack of anticipation of the attacks, generalizing their anticipatory anxiety, stress, and depressive symptoms [38]. They also show loss of self-confidence and trust in others and present more problems in academic achievement [39] and more isolation and feelings of loneliness [40], as they are frequently victims of traditional bullying at the same time (Gini et al., 2018).

### 1.3. The Present Study

Previous scientific literature has mainly focused on analyzing, on the one hand, the negative consequences of harassment for the victims [41] and, on the other hand, the risk factors that explain the development of this type of behavior in the aggressors. Thus, the empirical evidence of the consequences of engaging in aggressive behavior for the aggressor’s psychological maladjustment is very scarce, and there is even less research on the individuals involved in harassment situations through the new technologies.

In view of these limitations, the objective of the present study was to analyze the extent to which violent peer behavior, both traditional and cybernetic, and victimization, both traditional and cybernetic, predict certain indicators of psychological maladjustment in adolescents, such as: self-concept (multidimensional), life satisfaction, feelings of loneliness, depressive symptomatology, perceived stress, social anxiety, cognitive empathy and emotional empathy, and emotional intelligence (emotional attention, emotional clarity, and emotion regulation). We analyzed these variables and their dimensions as a consequence of harassment and in the four roles: traditional aggressor, cyberaggressor, traditional victim, and cybervictim.

## 2. Materials and Methods

### 2.1. Participants

Participants in the study were 1318 adolescents (47% boys and 53% girls), aged between 11 and 18 years (M = 13.8, SD = 1.32), enrolled in four Compulsory Secondary Education (CSE) schools in the Andalusian, Aragonese, and Valencian communities. The distribution of students by academic level was balanced: 24.7% was enrolled in first grade of CSE, 27.3% in second grade 23.7% in third grade, and 24.3% in fourth grade. For sample selection, probabilistic sampling was carried out using as primary sampling units the urban geographic areas of the provinces of Alicante, Valencia, Seville, and Teruel, and as secondary units, the public institutes in each area. The grades or classrooms were not used as tertiary units, as all the students of the four courses of CSE in all the schools participated. The socioeconomic level of the areas and schools was average. Approximately one fourth of the parents of the participating students had primary education, one fourth had secondary education, one fourth had high school studies, and one fourth had university studies. Most of the parents had a paid job outside the home: 86.7% of the fathers and 69.5% of the mothers.

### 2.2. Instruments

Victimization Scale of Mynard and Joseph [42]. This 22-item instrument measures three dimensions on a four-point Likert-type scale (1 = never, 4 = always): Relational Victimization (e.g., “A classmate told others not to be my friends”), Overt Physical Victimization (e.g., “A classmate beat me up”), and Overt Verbal Victimization (e.g., “A classmate picked on me”). The Cronbach alpha of the global scale in the sample was 0.94, and for the three dimensions, it was 0.92, 0.68, and 0.88, respectively.

Scale of Violent Behavior of Little et al. [43]. This scale consists of 25 items concerning participation in aggressive peer behavior in school population, rated on a four-point Likert-type scale ranging from 1 (never) to 4 (always). The scale measures two types of aggressive behavior (manifest or direct, and relational or indirect) and three functions of violence (pure, reactive, and instrumental), producing six dimensions of aggression: Pure Overt (e.g., “I’m the kind of a person who fights with others”), Reactive Overt Violence (e.g., “When someone hurts or injures me, I hit him”), Instrumental Overt Violence (e.g., “I threaten others to get what I want”), Pure Relational Violence (e.g., “I’m the kind of person who gossips and spreads rumors about others”), Reactive Relational Violence (e.g., “When someone annoys me, I ignore them or stop talking to them”), and Instrumental Relational Violence (e.g., “To get what I want, I despise others”). The Cronbach alpha of the six dimensions in the present study ranged between 0.63 and 0.83.

Self-concept Scale Form-5 (AF5) of García & Musitu [44]. This 24-item scale measures four dimensions of self-concept (six items per dimension): Academic (e.g., “I work a lot in class”), Social (e.g., “I have trouble talking to strangers”), Family (e.g., “I am very happy at home”), and Physical (e.g., “I take care of myself”). The response scale ranges between 1 (strongly disagree) and 9 (strongly agree). The Cronbach alpha of the global scale in this sample was 0.89, and for the four dimensions was 0.91, 0.76, 0.87, and 0.80, respectively.

“Test de Empatía Cognitiva y Afectiva” (TECA, Cognitive and Affective Empathy Test) of López-Pérez et al. [45]. This 33-item scale assesses on a five-point Likert-type scale (1 = never, 5 = always) the cognitive and affective components of empathy which, in turn, are subdivided into four dimensions. The component of cognitive empathy includes the dimensions of Perspective-taking (e.g., “I try to understand my friends better by looking at situations from their perspective”) and Emotional Comprehension (e.g., “I realize when someone tries to hide their true feelings”). The affective empathy component includes the parts of Empathic Stress (e.g., “Unless it is something very serious, I find it hard to cry about what happens to others”) and Empathic Joy (e.g., “When something good happens to someone, I feel happy”). The Cronbach alpha of the global scale obtained in the present sample was 0.83, and for the subscales, it ranged between 0.65 and 0.78.

Trait Meta-Mood Scale (TMMS) of Salovey et al. [46], adapted to Spanish by Fernández-Berrocal et al. [47]. This scale consists of 22 items with five Likert-type response options (1 = totally disagree; 5 = totally agree), and three dimensions: Emotional Attention (e.g., “I pay close attention to how I feel”), Emotional Clarity (e.g., “I feel clear about my feelings”), and Emotion Regulation (e.g., “When I am angry, I try to change my mood”). The internal consistency (Cronbach alpha) of the scale was 0.91, and for the three dimensions, it was 0.91, 0.86, and 0.87, respectively.

Perceived Stress Scale of Cohen et al. [48]. This scale consists of 14 items that evaluate on a four-point Likert format (1 = never, 4 = always) the degree to which the subject has experienced certain situations as stressful in the past month (e.g., “In the past month, I felt I could not control the most important things of my life”). The internal consistency index (Cronbach alpha) in the present sample was 0.66.

Satisfaction with Life Scale of Diener et al. [49], adaptation of Atienza et al. [50]. This instrument contains five items that provide a general index of subjective perceived well-being (e.g., “I’m not happy with my life”). The items are rated on a four-point Likert-type scale ranging from 1 (strongly disagree) to 4 (strongly agree). The internal consistency index (Cronbach alpha) in the present sample was 0.78.

UCLA Loneliness Scale of Russell et al. [51], adapted to Spanish by Expósito and Moya [52]. This scale consists of 20 items with a four-point Likert-type response option (1 = never, 4 = always), which provide a general measure of feelings of loneliness (e.g., “How often do you feel isolated from others?”). The scale presented a Cronbach alpha reliability in this study of 0.92.

Center for Epidemiologic Studies Depression Scale (CES-D; [53]; bidirectional translation). This scale is made up of 20 items that assess symptomatology related to depressed mood on a four-point Likert-type scale ranging from 1 (never) to 4 (always) The CESD provides a general rate of depressive mood that does not evaluate depression itself, but the symptomatology that is usually associated with it (e.g., “For the past week, I’ve felt sad”). The reliability of the instrument according to the Cronbach alpha obtained in the present sample was 0.84.

Social Anxiety Scale for Adolescents (SAS-A; La Greca & López, [54]; adaptation of Olivares et al. [55]). Through 22 items with a five-point Likert-type scale (1 = never, 4 = always), it assesses adolescent social anxiety responses in the context of their interpersonal relationships through three dimensions: fear of negative evaluation (e.g., “I am concerned about being evaluated by others”), avoidance and social anxiety in new situations (e.g., “I get nervous when they introduce me to strangers”), and avoidance and social anxiety in general (e.g., “I’m ashamed even when I’m with people I know well”). The global Cronbach alpha of the present study was 0.91, and for the three dimensions, it was 0.87, 0.81, and 0.78, respectively.

### 2.3. Procedure

The data of this work were collected as part of a broader study on psychological adaptation in adolescence, prior authorization of the pertinent educational authorities at the local, regional, and state level, as well as by the Evaluation Body of Projects of the University headquarters from which the project is directed. For data collection, firstly, an initial telephone contact was established with the directors of the schools, followed by a meeting with all the teaching staff in which the objectives of the study and the procedure to be followed for data collection were reported. After the collaboration of the schools was agreed on, an explanatory letter was sent to the parents requesting their consent for the participation of their children in the study. After obtaining parental consent (about 1% of the parents did not authorize their child to participate), the administration of the instruments was carried out by a group of previously trained researchers, in normal school hours in the habitual classrooms of the students, who participated voluntarily. Their privacy and the confidentiality of the information provided were guaranteed.

## 3. Results

### 3.1. Statistical Analysis

In order to examine and quantify the predictive capacity of aggressive behavior and victimization for the adaptation variables of interest, we performed binary logistic regression analysis using the forward stepwise regression procedure based on the Wald statistic. Logistic regression analysis estimates the probability of an event or outcome occurring in the presence of the predictor variable. This probability is estimated by the Odds Ratio (OR) statistic. A higher OR value indicates that the increase of the independent variable leads to an increase in the probability of the event occurring. In contrast, an OR value lower than 1 indicates that an increase in the independent variable leads to a decrease in the probability of the occurrence of the event [56].

To carry out these analyses, the criterion variables (each one of the indicators of psychological maladjustment) were dichotomized according to percentiles 25 and 75 to identify the high or low presence of the construct. The proportion of cases correctly classified by the logistic models ranged from 69.8% (avoidance and social anxiety in new situations) to 79.3% (life satisfaction) in the sample of traditional victims, between 69.4% (avoidance and social anxiety in new situations) and 77.8% (life satisfaction) in the sample of cybervictims, between 69.5% (depressive symptomatology) and 77.8% (life satisfaction) in the sample of traditional aggressors, and between 69.5% (depressive symptomatology) and 77.8% (life satisfaction) in the sample of cyberaggressors.

### 3.2. Traditional and Cybernetic Aggressive Behavior as Predictors of Psychological Maladjustment

Table 1 and Table 2 show the logistic regression for the probability of low and high scores in the maladjustment indicators for traditional aggressors. The first ORs presented in Table 1 indicate that the probability of presenting low academic and family self-concept increased 3.34 and 1.91 times, respectively, for each point that aggressive behavior increased. The dimensions of empathy called perspective-taking, empathic stress, and empathic joy showed an OR of 2.93, 1.39, and 2.00, respectively. Regarding the dimensions of emotional intelligence, low scores in emotion regulation, with an OR of 1.74, were more likely. For each point of increase of aggressive behavior, a statistically significant probability of increase of 3.72 points in low life satisfaction was also observed.

The ORs presented in Table 2 indicate that the probability of presenting high perception of loneliness, depressive symptomatology, and perceived stress increased by 1.66, 3.15 and 1.78 points, respectively, in traditional aggressors. A similar increase was observed in the social anxiety variables called fear of negative evaluation (OR = 2.25) and avoidance and social anxiety in general (OR = 2.07).

The three most important indicators of psychological maladjustment in traditional aggressors were, in this order: low life satisfaction, low academic self-concept, and high depressive symptomatology.

Table 3 and Table 4 show the logistical regressions calculated for cyberaggressors. The results presented in Table 3 indicate that the probability of presenting low academic self-concept and low family self-concept increased 2.61 and 2.02 times, respectively, for each point of increase of aggressive behavior. Statistically significant probabilities were also obtained for low scores in the four dimensions of the empathy construct, with values of OR between 1.35 and 2.14 in online aggressors, as well as low life satisfaction, with an OR 2.61.

The ORs presented in Table 4 indicate that the probability of presenting high loneliness, depressive symptomatology, and perceived stress increased by 1.57, 2.19, and 1.60 points, respectively. A similar increase was observed in the dimensions of social anxiety, fear of negative evaluation (OR = 2.06), and avoidance and social anxiety in general (OR = 1.88). The dimensions avoidance and social anxiety in new situations and emotional attention were not significant.

The three most important indicators of psychological maladjustment in cyberaggressors were the same as for traditional aggressors, that is, low life satisfaction, low academic self-concept, and high depressive symptomatology. However, the ORs associated with these three indicators were lower in the cyberaggressor group.

### 3.3. Traditional and Cybernetic Victimization as Predictors of Psychological Maladjustment

Table 5 and Table 6 show the logistic regression for the probability of low and high scores on the indicators of maladjustment in traditional victims. The results presented in Table 5 indicate that the probability of presenting low self-concept increases for all four dimensions (academic, family, physical, and social) with values of OR ranging between 1.48 and 2.08, for each point of increase in traditional victimization. In emotional intelligence, low scores in the dimensions of emotional clarity (OR = 1.45) and emotion regulation (OR = 1.68) were also observed to be more likely. The OR associated with low life satisfaction obtained a statistically significant value of 4.44 in this group.

The data presented in Table 6 indicate that the probability of a high feeling of loneliness, as well as of depressive symptomatology and perceived stress, increased by 4.29, 5.42, and 3.30 points, respectively, in traditional victims. The dimension of emotional intelligence that refers to the degree of attention to one’s own emotions also showed an increase of 1.40 times among the victims. Finally, a notable increase was observed in the three dimensions of social anxiety: fear of negative evaluation (OR = 4.09), avoidance and social anxiety in new situations (OR = 1.86), and avoidance and social anxiety in general (OR = 3.34).

The three indicators of psychological maladjustment with greater negative influence on traditional victims were, in this order: high depressive symptomatology, low life satisfaction, and high feelings of loneliness.

Table 7 and Table 8 show the logistic regression for the probability of low and high scores on the maladjustment indicators in the group of cybervictims. The results of Table 7 indicate that the probability of presenting low self-concept also increased, in this case, for the four dimensions analyzed, presenting values of OR between 1.82 and 2.22. In emotional intelligence, low scores in the dimensions of emotional clarity (OR = 1.61) and emotion regulation (OR = 1.34) in cybervictims were also more likely to be predicted. A similar increase was observed in the probability of presenting low emotional comprehension of the variable empathy (OR = 1.33). The OR associated with low life satisfaction obtained a statistically significant value of 4.45 in this group.

The results presented in Table 8 show that the probability of experiencing a high feeling of loneliness, depressive symptomatology, and perceived stress increased by 3.48, 4.92, and 2.99 points, respectively, in cybervictims. A similar increase was observed in the dimensions of social anxiety, fear of negative evaluation (OR = 3.87), avoidance and social anxiety in new situations (OR = 1.65), and avoidance and social anxiety in general (OR = 3.23).

The three most important indicators of psychological maladjustment in cybervictims were, in this order: high depressive symptomatology, low life satisfaction, and high social anxiety due to fear of negative evaluation.

## 4. Discussion

The objective of the present study was to analyze the extent to which violent peer behavior, both traditional and cybernetic, and victimization, both traditional and cybernetic, predict certain indicators of psychological maladjustment in adolescence. The results have shown that the victims in general present greater maladjustment in most of the indicators analyzed and with higher values.

In a more detailed analysis by variables and dimensions, it was observed that victims and aggressors both show negative self-perceptions in some domains, although the victims’ self-concept seems to be more damaged. All the victims had a higher probability of informing of a negative self-concept in the four dimensions (family, academic, physical, and social). The results of previous studies, such as the one carried out by Piñuel and Oñate [57], also found that the students identified as victims perceived a negative effect of harassment on all the dimensions of self-concept. Subsequent research has continued to observe a negative self-concept at the multidimensonal level both in traditional victims [31] and in cybervictims [4].

The aggressors, however, did not report having a more negative self-concept in the physical area (this is precisely the most affected dimension in the victims). Traditional aggressors do seem to have a more negative social self-image as a result of their behavior (an association not observed in cyberaggressors). This result suggests that traditional aggressors’ social interactions with their classmates and peers may be more negatively affected than the interactions of cyberaggressors, who harass others more discreetly and are less exposed to observation and, hence, to peer evaluation. However, both traditional and cyberaggressors showed a more negative self-image regarding the academic and family domains.

In studies with adolescents presenting violent behavior, it has been found that their family environment tends to be characterized by a poor parent-child emotional bond [58], the adolescents’ perception of a significant lack of support [59], and the presence of frequent family conflicts [60]. The results of this study indicate that adolescents’ perception of family dysfunction and a poor self-concept in this environment, can, in fact, be aggravated by their behavior toward their peers at school. In parallel, our results indicate a negative academic self-concept, in line with other works such as that developed by Taylor et al. [18] with traditional aggressors, in which they identified less positive self-perceptions in these students with regard to their academic skills, as well as more negative attitudes towards studies and the teachers. The evidence of a multidimensional self-concept in cyberaggressors is an important contribution of the present study.

The results related to empathic capacity showed significant OR values in both types of aggressors, who, in general, showed lower levels both in the emotional and the cognitive dimension of empathy. These results are consistent with those found in previous investigations that indicate that school aggressors tend to show a deficit in empathic capacity, which is why they have difficulties identifying with the pain of the classmate whom they are victimizing [61]. In the same line, Hinduja and Patchin [62] concluded that cyberaggressors also have low empathy because their indirect violence through new technologies does not allow them to directly appraise the consequences of their behavior for the victim. The results of the present study suggest that the cognitive and emotional empathy of aggressors, both traditional and cybernetic, can, in turn, be diminished by their repetitive violent behavior towards their victim, perhaps through a process of desensitization or moral disengagement, as some authors have pointed out [63].

Either because of their aggressive behavior or because of their situation of harassment, all four roles analyzed seem to suffer an increase in their feelings of loneliness, depressive symptomatology, and stress, as well as a decrease of their general life satisfaction. However, the OR values are much higher in the case of the victims (in both contexts) compared to the aggressors (in both contexts). This result coincides with that observed in previous works with respect to traditional and cybernetic victims (e.g. [64,65,66]) in which the emotional maladjustment involved by experiencing a situation of peer abuse has been systematically verified. As suggested, social or emotional maladjustment is also a relevant consequence, as it seems that the victims are increasingly isolated [67,68] and perceive an increasing degree of rejection or non-acceptance by their peers [69]. Studies of aggressors have identified social isolation, depression, stress, and dissatisfaction with life as risk factors (e.g., [12,13,70]). In addition, the findings of this study suggest that aggressors’ behavior aggravates their emotional maladjustment, although to a lesser extent than in the case of the victims. This fact is confirmed both for traditional school aggression and for cyberaggression.

With regard to social anxiety, the four roles analyzed showed an increase in the anxiety experienced in general situations of social interaction, although the ORs associated with the traditional and cybernetic victims were higher than those obtained by the aggressors. This same result was observed with regard to the dimension of social anxiety called fear of negative evaluation, which showed an increase in all the roles, but more marked in all the victims. Both groups of victims also showed an increase in anxiety in new social situations but, in this case, the result was not significant in the aggressors. This suggests that traditional victims and cybervictims could be developing generalized negative expectations about their harassment situations toward new social interactions in other contexts or with other people. Studies on social anxiety in victims have been mainly confined to victims of traditional school harassment, with conclusions in the line of those contributed in this work, which allow extending the findings to the victims of cybernetic harassment: the loss of self-confidence and the sense of suffering and insecurity provoked by harassment lead to an increase in anticipatory anxiety and the avoidance of new situations of exposure to social contact [38].

Research on social anxiety in aggressors is even scarcer. In some studies with cyberaggressors, high levels of social anxiety have been observed, which the authors explain by the lack of social skills to interact with the peer group, which increases the likelihood that these people will make more use of the social networks and will attack others through technological means, thus escaping the anxiety caused by direct social contact [71,72]. Our results are in line with what was pointed out by these authors, as indeed the aggressors reported an increase of their fear of negative evaluation and of general social anxiety.

Finally, with regard to the emotional intelligence variable, the results of the present study have found that victims, in general, show a worsening in all the dimensions of this construct, that is: more emotional attention (especially the victim of traditional harassment), less clarity or emotional comprehension, along with a worse self-regulation of their own emotions. These findings are consistent with the empirical evidence on emotional intelligence in victimized persons in general, which indicates that the degree of emotional confusion to which they are subjected (low emotional clarity) is usually associated with high emotional attention because, when they feel emotionally confused, they must devote more cognitive resources to understand what is happening, whereas people with emotional clarity could devote those resources to selecting better strategies to cope with the problematic situation [73]. In fact, this is exactly what seems to happen with victims of both traditional and cybernetic school bullying: an emotional imbalance that prevents them from concentrating on choosing and using adaptive coping strategies [74], which coexists with the low regulation of their own emotions and, thus, a greater difficulty to return to a situation of well-being and emotional equilibrium [37].

The results obtained with respect to emotional intelligence in the groups of aggressors showed a worsening in a single dimension of the construct: emotion regulation, particularly in traditional aggressors. This result confirms the findings of other works that have reported that adolescents who presented more intense and frequent facial expressions of hostility and anger were less able to regulate and adequately manage their emotions, aspects that are present in students with violent behavior [22,75,76]. In fact, several previous studies have shown lower general levels of emotional intelligence in schoolchildren with a tendency to show aggressive behaviors [19,20,76,77], but there are hardly any referents of multidimensional emotional intelligence in aggressors, and even fewer in cyberaggressors. In this study, cyberaggressors did not show a worsening in any dimension of emotional intelligence or in emotion regulation. A possible explanation, besides the results obtained in empathy for this group, lies in the fact that exerting aggression indirectly may be associated with a process of desensitization or moral disengagement from the victim [63], which does not unbalance the aggressor’s emotions (a more uncontrollable aspect in a face-to-face aggression with the victim present).

Despite the scientific contributions of this study highlighted in the preceding paragraphs, this work also presents some limitations that the authors recognize and that we believe should be rectified in future works. First, the results obtained cannot be generalized to students of other educational levels, such as Primary Education, where problems of school aggression and of harassment through new technologies are also found in children, who are acquiring electronic devices at an early age [78]. Secondly, the data of this research are based exclusively on measures of self-report, a fact that can introduce biases derived from the participants’ social desirability, although previous studies have confirmed the reliability and validity of this type of instrument for the measurement of risk behaviors in adolescents [79]. Finally, the cross-sectional nature of the data precludes the establishment of causal relationships between the variables, so that the direction of the potential influences between them must be interpreted cautiously.

## 5. Conclusions

School violence has negative consequences for all involved, both aggressors and victims. Victims and aggressors both show negative self-perceptions in some domains, although the victims’ self-concept seems to be more damaged. All the victims had a higher probability of informing of a negative self-concept in the four dimensions (family, academic, physical, and social). The aggressors, however, did not report having a more negative self-concept in the physical area (this is precisely the most affected dimension in the victims). However, both traditional and cyberaggressors showed a more negative self-image regarding the academic and family domains. With regard to the empathic capacity, both types of aggressors showed, in general, lower levels both in the emotional and the cognitive dimension of empathy. Furthermore, all four roles analyzed showed an increase in the anxiety experienced in general situations of social interaction, although anxiety in traditional and cybernetic victims was even higher than in the aggressors. With regard to emotional intelligence, the findings of the present study let us conclude that the victims, in general, show a worsening in all dimensions of this construct, that is: more emotional attention (especially the victim of traditional harassment), less clarity or emotional comprehension, along with a worse self-regulation of their own emotions. With respect to emotional intelligence in the aggressors, they indicated a worsening in a single dimension of the construct: emotion regulation, particularly in traditional aggressors. A possible explanation, besides the results obtained in empathy for this group, lies in the fact that exerting aggression indirectly may be associated with a process of desensitization or moral disengagement from the victim, which does not unbalance the aggressor’s emotions.

Thus, taken together, the results of this work contribute to our knowledge of the influence of the role played in a bullying situation, as victim or aggressor, and depending on the context -school or online- in the psychological adjustment of the person involved. Taking into consideration the great overlap in roles in bullying and cyberbullying among classmates at school [27,28,29,30], together with the amount of time that children spend at the educational institution, there is no doubt that the school is an important context for preventive efforts designed to promote the wellness of at-risk students [80] through socio-emotional learning programs that contribute to the development of students’ social skills and emotional competence. These educational programs, either of prevention or intervention, should consider the different dimensions of psychological wellbeing analyzed in the present study, with special attention to emotional intelligence and empathy. They should contain activities that stimulate the development of knowledge of one’s own and others’ emotions, control of emotional impulses and behavior, and the development of positive social skills [81]. The promotion of the development of psychological and socioemotional skills, such as emotion regulation, in the main socialization institutions, such as family and school, may provide children and adolescents with the opportunity to achieve better psychosocial adjustment and prevent victimization situations [82,83]. In addition, developing the sensitivity of the school staff and the classmates to signs of victimization situations is a key aspect to guarantee peaceful coexistence.

## Figures and Tables

**Table 1:** Logistic regression with traditional aggression as a predictor of low scores.

		B	SE	Wald	*p*	OR	95% CI	
							Lower	Higher
Academic Self-concept	Aggression	1.20	0.22	29.27	0.00	3.34	2.15	5.17
	Constant	−2.85	0.32	79.56	0.00	0.05		
Family Self-concept	Aggression	0.64	0.21	9.04	0.00	1.91	1.25	2.91
	Constant	−2.06	0.30	45.13	0.00	0.12		
Physical Self-concept	Aggression	−0.05	0.22	0.06	0.80	0.94	0.60	1.47
	Constant	−0.99	0.31	9.96	0.00	0.37		
Social Self-concept	Aggression	0.42	0.22	3.68	0.05	1.52	0.99	2.35
	Constant	−1.83	0.31	34.29	0.00	0.15		
Perspective-taking	Aggression	1.07	0.22	23.62	0.00	2.93	1.90	4.52
	Constant	−2.72	0.31	73.40	0.00	0.06		
Emotional Clarity	Aggression	0.35	0.20	2.96	0.08	1.43	0.95	2.14
	Constant	−1.38	0.29	22.30	0.00	0.25		
Empathetic Stress	Aggression	−0.91	0.27	10.86	0.00	0.39	0.23	0.68
	Constant	−0.00	0.37	0.00	0.99	0.99		
Empathetic Joy	Aggression	0.69	0.21	10.48	0.00	2.0	1.31	3.04
	Constant	−2.08	0.30	46.78	0.00	0.12		
Life Satisfaction	Aggression	1.31	0.22	33.52	0.00	3.72	2.38	5.82
	Constant	−3.08	0.32	88.71	0.00	0.04		
Emotional Clarity	Aggression	0.31	0.22	2.05	0.15	1.37	0.89	2.11
	Constant	−1.61	0.31	27.02	0.00	0.19		
Emotion Regulation	Aggression	0.55	0.21	6.71	0.01	1.74	1.14	2.65
	Constant	−1.89	0.30	38.58	0.00	0.15		

B: estimated coefficient. SE: Standard Error of B. Wald: tests the unique contribution of each predictor. If the Wald statistic is significant (*p* < 0.05) then the parameter is useful to the model. OR: Odds Ratio, is the predicted change in odds for a unit increase in the predictor. 95% CI: The 95% confidence interval (CI), used to estimate the precision of the OR.

**Table 2:** Logistic regression with traditional aggression as a predictor of high scores.

		B	SE	Wald	*p*	OR	95% CI	
							Lower	Higher
Loneliness	Aggression	0.51	0.21	5.91	0.01	1.66	1.10	2.52
	Constant	−1.71	0.29	33.06	0.00	0.18		
Depressive Symptomatology	Aggression	1.14	0.21	28.34	0.00	3.15	2.06	4.81
	Constant	−2.41	0.30	62.44	0.00	0.08		
Perceived stress	Aggression	0.57	0.20	7.77	0.00	1.78	1.18	2.67
	Constant	−1.68	0.29	32.87	0.00	0.18		
Fear of negative assessment	Aggression	0.81	0.21	14.42	0.00	2.25	1.48	3.43
	Constant	−2.22	0.30	53.07	0.00	0.10		
Avoidance and social anxiety in new situations	Aggression	0.18	0.20	0.79	0.37	1.20	0.80	1.81
	Constant	−1.08	0.29	13.72	0.00	33		
Avoidance and social anxiety in general	Aggression	0.73	0.21	12.05	0.00	2.07	1.37	3.13
	Constant	−2.00	0.29	44.78	0.00	0.13		
Perceived emotional attention	Aggression	−0.21	0.23	0.79	0.37	0.81	0.51	1.28
	Constant	−0.84	0.32	6.63	0.01	0.43		

**Table 3:** Logistic regression with cyberaggression as a predictor of low scores.

		B	SE	Wald	*p*	OR	95% CI	
							Lower	Higher
Academic Self-concept	Aggression Constant	0.96	0.20	22.04	0.00	2.61	1.75	3.91
		−2.28	0.24	86.77	0.00	0.10		
Family Self-concept	Aggression Constant	0.70	0.19	12.73	0.00	2.02	1.37	2.99
		−1.97	0.23	69.36	0.00	0.13		
Physical Self-concept	Aggression Constant	0.32	0.19	2.71	0.09	1.38	0.94	2.05
		−1.44	0.23	37.62	0.00	0.23		
Social Self-concept	Aggression Constant	0.26	0.20	1.63	0.20	1.30	0.86	1.95
		−1.55	0.24	39.89	0.00	0.21		
Perspective-taking	Aggression Constant	0.76	0.20	14.64	0.00	2.14	1.45	3.17
		−2.10	0.23	77.24	0.00	0.12		
Emotional Comprehension	Aggression Constant	0.54	0.19	7.98	0.00	1.73	1.18	2.53
		−1.51	0.23	43.49	0.00	0.21		
Empathetic Stress	Aggression Constant	−1.03	0.36	8.26	0.00	0.35	0.17	0.71
		−.09	0.39	0.05	0.80	0.90		
Empathetic Joy	Aggression Constant	0.53	0.19	7.47	0.00	1.71	1.16	2.51
		−1.74	0.23	55.11	0.00	0.17		
Life Satisfaction	Aggression	0.96	0.20	22.05	0.00	2.61	1.75	3.90
	Constant	−2.35	0.24	91.93	0.00	0.09		
Emotional Clarity	Aggression Constant	0.17	0.21	0.70	0.40	1.19	0.79	1.79
		−1.38	0.24	31.36	0.00	0.25		
Emotion Regulation	Aggression Constant	0.13	0.21	.41	0.52	1.14	0.75	1.72
		−1.27	0.24	26.90	0.00	0.27		

**Table 4:** Logistic regression with cyberaggression as a predictor of high scores.

		B	SE	Wald	*p*	OR	95% CI	
							Lower	Higher
Loneliness	Victimization	0.45	0.19	5.37	0.02	1.57	1.07	2.30
	Constant	−1.52	0.23	43.27	0.00	0.21		
Depressive Symptomatology	Victimization	0.78	0.20	15.36	0.00	2.19	1.48	3.25
	Constant	−1.72	0.23	53.23	0.00	0.17		
Perceived Stress	Victimization	0.47	0.19	5.88	0.01	1.60	1.09	2.33
	Constant	−1.41	0.22	38.38	0.00	0.24		
Fear of negative assessment	Victimization	0.72	0.19	13.35	0.00	2.06	1.39	3.04
	Constant	−1.92	0.23	66.50	0.00	0.014		
Avoidance and social anxiety in new situations	Victimization	0.06	0.20	0.09	0.76	1.06	0.71	1.57
	Constant	−0.89	0.23	14.44	0.00	0.40		
Avoidance and social anxiety in general	Victimization	0.63	0.19	10.48	0.00	1.88	1.28	2.77
	Constant	−1.71	0.23	54.38	0.00	0.18		
Perceived Emotional Attention	Victimization	0.18	0.23	0.61	0.43	0.83	0.52	1.32
	Constant	−0.92	0.27	11.47	0.00	0.39		

**Table 5:** Logistic regression with traditional victimization as a predictor of low scores.

		B	SE	Wald	*p*	OR	95% CI	
							Lower	Higher
Academic Self-concept	Victimization Constant	0.39	0.13	8.92	0.00	1.48	1.14	1.92
		−1.80	0.22	66.02	0.00	0.16		
Family Self-concept	Victimization Constant	0.47	0.13	12.92	0.00	1.60	1.24	2.07
		−1.91	0.22	74.79	0.00	0.14		
Physical Self-concept	Victimization Constant	0.73	0.12	32.08	0.00	2.08	1.61	2.68
		−2.24	0.22	104	0.00	0.10		
Social Self-concept	Victimization Constant	0.56	0.13	17.70	0.00	1.75	1.35	2.27
		−2.14	0.22	89.76	0.00	0.11		
Perspective-taking	Victimization Constant	0.02	0.14	0.03	0.84	1.02	0.78	1.35
		−1.27	0.22	30.89	0.00	0.28		
Emotional Comprehension	Victimization Constant	−0.03	0.13	0.06	0.79	0.96	0.74	1.24
		−0.84	0.21	15.72	0.00	0.43		
Empathetic Stress	Victimization Constant	0.05	0.14	0.14	0.70	1.05	0.80	1.39
		−1.32	0.22	33.52	0.00	0.26		
Empathetic Joy	Victimization Constant	−0.03	0.13	0.05	0.82	0.96	0.73	1.27
		−1.08	0.22	23.27	0.00	0.33		
Life Satisfaction	Victimization	1.49	0.14	108.29	0.00	4.44	3.35	5.88
	Constant	−3.68	0.25	215.16	0.00	0.02		
Emotional Clarity	Victimization Constant	0.37	0.13	8.02	0.00	1.45	1.12	1.89
		−1.77	0.22	63.90	0.00	0.16		
Emotion Regulation	Victimization Constant	0.52	0.13	16.09	0.00	1.68	1.30	2.18
		−1.95	0.22	79.11	0.00	0.14		

**Table 6:** Logistic regression with traditional victimization as a predictor of high scores.

		B	SE	Wald	*p*	OR	95% CI	
							Lower	Higher
Loneliness	Victimization	1.45	0.14	108.49	0.00	4.29	3.26	5.64
	Constant	−3.35	0.23	196.73	0.00	0.03		
Depressive Symptomatology	Victimization	1.69	0.14	135.56	0.00	5.42	4.08	7.21
	Constant	−3.54	0.24	210.19	0.00	0.02		
Perceived Stress	Victimization	1.19	0.13	80.63	0.00	3.30	2.54	4.29
	Constant	−2.79	0.22	154.15	0.00	0.06		
Fear of negative assessment	Victimization	1.40	0.14	101.87	0.00	4.09	3.11	5.38
	Constant	−3.38	0.24	196.78	0.00	0.03		
Avoidance and social anxiety in new situations	Victimization	0.62	0.12	24.73	0.00	1.86	1.45	2.38
	Constant	−1.81	0.20	75.01	0.00	0.16		
Avoidance and social anxiety in general	Victimization	1.20	0.13	80.95	0.00	3.34	2.57	4.35
	Constant	−2.93	0.22	163.84	0.00	0.05		
Perceived emotional attention	Victimization	0.33	0.13	6.50	0.01	1.40	1.08	1.81
	Constant	−1.65	0.22	57.00	0.00	0.19		

**Table 7:** Logistic regression with cybervictimization as a predictor of low scores.

		B	SE	Wald	*p*	OR	95% CI	
							Lower	Higher
Academic Self-concept	Victimization Constant	0.59	0.15	15.74	0.00	1.82	1.35	2.44
		−1.93	0.20	90.50	0.00	0.14		
Family Self-concept	Victimization Constant	0.80	0.15	27.55	0.00	2.22	1.65	3.00
		−2.17	0.20	112.02	0.00	0.11		
Physical Self-concept	Victimization Constant	0.66	0.15	19.60	0.00	1.94	1.44	2.60
		−1.90	0.20	90.04	0.00	0.14		
Social Self-concept	Victimization Constant	0.75	0.15	24.63	0.00	2.13	1.58	2.87
		−2.21	0.20	113.94	0.00	0.11		
Perspective-taking	Victimization Constant	0.15	0.16	0.96	0.32	1.17	0.85	1.60
		−1.42	0.21	45.20	0.00	0.24		
Emotional Comprehension	Victimization Constant	0.29	0.14	3.82	0.05	1.33	0.99	1.78
		−1.25	0.19	41.15	0.00	0.28		
Empathetic Stress	Victimization Constant	−0.10	0.17	0.37	0.54	0.89	0.63	1.27
		−1.11	0.22	23.80	0.00	0.32		
Empathetic Joy	Victimization Constant	0.26	0.15	3.05	0.08	1.30	0.96	1.77
		−1.46	0.20	51.54	0.00	0.23		
Life Satisfaction	Victimization	1.49	0.17	74.07	0.00	4.45	3.17	6.26
	Constant	−3.15	0.23	182.90	0.00	0.04		
Emotional Clarity	Victimization Constant	0.47	0.15	9.90	0.00	1.61	1.19	2.16
		−1.78	0.20	76.90	0.00	0.16		
Emotion Regulation	Victimization Constant	0.29	0.15	3.66	0.04	1.34	0.99	1.81
		−1.49	0.20	53.78	0.00	0.22		

**Table 8:** Logistic regression with cybervictimization as a predictor of high scores.

		B	SE	Wald	*p*	OR	95% CI	
							Lower	Higher
Loneliness	Victimization	1.24	0.16	56.69	0.00	3.48	2.51	4.82
	Constant	−2.58	0.21	138.81	0.00	0.07		
Depressive Symptomatology	Victimization	1.59	0.18	74.81	0.00	4.92	3.43	7.07
	Constant	−2.82	0.23	141.80	0.00	0.05		
Perceived Stress	Victimization	1.09	0.16	46.21	0.00	2.99	2.18	4.10
	Constant	−2.26	0.21	113.79	0.00	0.10		
Fear of negative assessment	Victimization	1.35	0.16	64.11	0.00	3.87	2.78	5.40
	Constant	−2.81	0.22	156.64	0.00	0.06		
Avoidance and social anxiety in new situations	Victimization	0.50	0.14	11.69	0.00	1.65	1.23	2.20
	Constant	−1.45	0.19	56.15	0.00	0.23		
Avoidance and social anxiety in general	Victimization	1.17	0.16	51.79	0.00	3.23	2.34	4.45
	Constant	−2.47	0.21	131.26	0.00	0.08		
Perceived emotional attention	Victimization	0.13	0.15	0.72	0.39	1.14	0.83	1.56
	Constant	−1.29	0.20	38.81	0.00	0.27		
